# RadarTCN: Lightweight Online Classification Network for Automotive Radar Targets Based on TCN

**DOI:** 10.3390/s24092813

**Published:** 2024-04-28

**Authors:** Yuan Li, Mengmeng Zhang, Hongyuan Jing, Zhi Liu

**Affiliations:** 1 School of Electrical and Control Engineering, North China University of Technology, Beijing 100144, China; liyuan104@mail.ncut.edu.cn; 2 School of Information, North China University of Technology, Beijing 100104, China; 3 College of Robotics, Beijing Union University, Beijing 100101, China; jqrhongyuan@buu.edu.cn

**Keywords:** automotive radar image, target classification, TCN, range-angle, range-Doppler

## Abstract

Automotive radar is one of the key sensors for intelligent driving. Radar image sequences contain abundant spatial and temporal information, enabling target classification. For existing radar spatiotemporal classifiers, multi-view radar images are usually employed to enhance the information of the target and 3D convolution is employed for spatiotemporal feature extraction. These models consume significant hardware resources and are not applicable to real-time applications. In this paper, RadarTCN, a novel lightweight network, is proposed that achieves high-accuracy online target classification using single-view radar image sequences only. In RadarTCN, 2D convolution and 3D-TCN are employed to extract spatiotemporal features sequentially. To reduce data dimensionality and computational complexity, a multi-layer max pooling down-sampling method is designed in a 2D convolution module. Meanwhile, the 3D-TCN module is improved through residual pruning and causal convolution is introduced for leveraging the performance of online target classification. The experimental results demonstrate that RadarTCN can achieve high-precision online target recognition for both range-angle and range-Doppler map sequences. Compared to the reference models on the CARRADA dataset, RadarTCN exhibits better classification performance, with fewer parameters and lower computational complexity.

## 1. Introduction

With the development of intelligent driving technology, cars equipped with intelligent driving systems are becoming more and more popular with consumers. Millimeter wave radar, which is not disturbed by light, haze, rain, and snow, can detect obstacle targets in a variety of complex traffic scenarios and has become an important sensor of intelligent driving systems. At present, automotive radar mainly relies on sparse points cloud technology to detect targets. Points cloud data are obtained through traditional radar signal processing algorithms such as constant false alarm rate (CFAR) [1] and DBSCAN clustering algorithm [2] and the target is represented by as few echo points as possible, which only carry the result information obtained by signal processing. However, compared with the original radar data, the sparse points cloud of radar has some loss in semantic information [3].

Radar raw data, which are the information obtained by collecting received signals from the analog-to-digital conversion module in the radar baseband circuit section, contain rich semantic information about targets. In the process of target detection, for the convenience of processing, Fourier transform is generally used to convert the radar raw data from time-domain signals to frequency–domain signals. The transformed results are usually presented in the form of radar images such as range-Doppler map (RDM), range- angle map (RAM), and angle-Doppler map (ADM). By integrating RDM, RAM, and ADM data, multi-view spectral data of range-angle-Doppler (RAD) can be obtained [4].

In recent years, detection and recognition algorithms based on convolutional neural networks (CNN) have achieved great success in the field of imaging, prompting researchers to experiment with using neural network models to extract target feature information from radar images. The characteristics of the target, such as RCS, Doppler, and phase, are represented on RDM and RAM radar images. Based on this type of feature information, target detection and recognition can be achieved [5,6,7]. Ref. [8] uses CNN to extract target Doppler shift information from radar time–frequency diagrams for detection and classification. Refs. [9,10] also propose target classification methods based on micro-Doppler characteristics. However, these methods rely on a single radar feature for classification and detection.

Compared to optical images, the application scope of radar raw data is quite limited and its acquisition and annotation process is also relatively tricky. Furthermore, the complicated processing of radar signals makes obtaining RAM, RDM, and RAD data a time-consuming and labor-intensive task. However, since 2019, datasets containing millimeter-wave radar raw information have been successively made public, with the most representative ones including the CARRADA dataset [11], CRUW dataset [12], and RADDet dataset [13]. These radar datasets, which encompass radar spectrum data, ground truth labels, and corresponding camera data, provide researchers with great convenience. These datasets have propelled the advancement of detection and recognition algorithm technologies based on radar spectrum data.

RDM characterizes the spatial distribution of target distances and the frequency distribution of Doppler dimensions. By utilizing deep learning models, Doppler and distance features can be extracted from RDM to achieve target classification [14,15,16,17]. RAM characterizes the distribution of target echoes along the distance axis and the azimuthal angle axis. If the resolution of RAM is sufficiently high, it closely approximates a spectrum comparable to an optical image, enabling the implementation of radar image semantic segmentation and target location detection based on RAM [18,19,20,21]. RAD comprises echo information derived from multi-view of the radar signal, abundant in semantic information. More detailed target feature extraction can be performed based on RAD for target classification and detection [22,23,24,25,26]. Limited by antenna resolution, automotive radars struggle to distinguish side-by-side moving targets, such as large trucks and small cars. Studies like [24,27] introduce time-domain dimension information and utilize dynamic changes in consecutive frames to distinguish targets, bypassing the limitations of radar antenna resolution and achieving higher classification metrics. In real-world scenarios, automotive radars are required to perform online detection. Ref. [27] constructs a causal network that utilizes RAD data from previous moments to achieve online classification and detection. However, the baseband signal processing resources of automotive radars are limited and the computation process to obtain multi-view radar data from raw data is very time-consuming and labor-intensive. Additionally, complex models will increase the demand for hardware computing power and power consumption, which is very challenging for automotive radars with strict cost and size constraints. The model in [24] has a parameter scale of 106 M, which requires high hardware resources and faces difficulties in application.

This paper proposes a lightweight network capable of online target classification, named RadarTCN. RadarTCN accomplishes classification tasks using only single-view radar images (RAM or RDM). The data dimensionality of RAM or RDM is significantly lower than that of multi-view data RAD, which not only reduces the computational load of the model but also effectively lowers the computational complexity of the radar signal processing unit. The main contributions of this paper are as follows:

- A 2D convolution module is utilized to capture spatial information and 3D-TCN is employed to extract temporal features from radar image sequences. This strategy, which separates the processing of spatial and temporal information, effectively prevents information loss that could arise when a 3D convolution network is used to simultaneously handle both types of information;
- Given the characteristics of target echoes exhibiting a converging state on radar images and reaching local peak power values, this paper utilizes multi-layer max pooling technology in the spatial information processing module to effectively reduce data dimensionality and computational complexity, significantly decreasing the demand for hardware resources;
- This paper innovatively applies TCN to the field of automotive radar classification. The structure of the 3D-TCN network is improved and the lightweight design of the model is achieved by pruning the residual network, effectively decreasing the network’s parameters and GMACS (Giga Multiply-Accumulate Operations per Second). Meanwhile, this paper employs causal convolution to construct a temporal feature extraction module, which effectively extracts temporal features from radar data, enabling online target classification by the model;
- A dataset processing method that simulates radar’s online operation is proposed. The open dataset CARRADA [11] is selected for model training and evaluation. According to the processing flow of radar’s real-time data acquisition and transmission to the classification module, the CARRADA dataset is re-split and repackaged to form an image sequence dataset, aiming to provide a more realistic simulation of radar’s online operation.

The remainder of this paper is organized as follows. Section 2 introduces the semantic information of radar images and reviews the research progress of target feature extraction techniques based on radar data sequences. Section 3 proposes the RadarTCN network architecture, elaborates on the implementation scheme and advantages of the spatiotemporal feature extraction network, and details the dataset processing methods and model training approaches. Experimental results and discussions are presented in Section 4. Finally, Section 5 draws the conclusions of this paper together.

## 2. Related Work

### 2.1. Semantic Features of Radar Echo

Automotive radar is an active detection device. The radar transmitting antenna radiates frequency-modulated continuous waves (FMCW) outwards. When the transmitted signal encounters a target, it reflects part of the echo signal back. The distribution of the target-reflected echo on the radar image is related to the backscatter characteristics of the target. The scattering characteristics of the target are effective evidence for target identification based on factors such as the shape and material of the target.

The size of traffic targets generally far exceeds the resolution of automotive radars. The larger the target size, the more scattering points it exhibits on the radar image, with each scattering point representing the echo of an independent scatterer. Traffic targets are typically complex reflectors and their scattering cross-section is a complex function of viewing angle and radiated signal wavelength. Complex reflectors can be approximately decomposed into multiple independent scatterers, with no interaction between the various parts. In this case, the total scattering cross-section *σ* of the target is the vector sum of the cross-sections of the individual scatterers [28].

(1) $$\sigma ={\left|\underset{k}{\sum }\sqrt{\sigma_{k}}\exp\left(\frac{j4\pi R_{k}}{\lambda }\right)\right|}^{2}$$

where $\sigma_{k}$ is the cross-sectional area of the *k*th scatterer and $R_{k}$ is the distance between the *k*th scatterer and the radar. The shape of the target determines the distribution of its scatterers. With a fixed incident angle, the distribution of scatterers determines the distribution characteristics of the target scattering points on the radar image [29].

The Doppler shift generated by radar echo data in the time domain also contains abundant target feature information [30]. Between consecutive detection periods, changes in the range dimension of the target on the radar image reflect speed information, while changes in the Doppler dimension reflect feature information such as acceleration and micro-motion characteristics.

(2) $$f_{d}=\frac{1}{\lambda }\cdot \frac{d\left(2R+\Delta r\right)}{dt}$$

where $f_{d}$ represents the target Doppler frequency, λ represents the carrier wavelength, R represents the distance to the target at the initial moment of the detection period, and Δr represents the position change within one detection period. If the target moves at a constant speed, Δr is a fixed value and $f_{d}$ remains constant; if the target is in a non-uniform motion state, $f_{d}$ is a varying value and this type of phenomenon is characterized in the RDM.

### 2.2. Radar Image Sequence Feature Extraction

Three-dimensional CNNs incorporate the time dimension, enabling them to process sequential data. Xiangyu Gao et al. [24] propose a radar multi-perspective convolutional neural network (RAMP-CNN) to detect the location and category of targets from sequences of range-Doppler-angle maps. RAMP-CNN comprises three convolutional autoencoders (CAEs) consisting of 3D CNN layers and 3D transposed convolution layers, which process RAM, RDM, and ADM sequences separately to extract temporal information. Yizhou Wang et al. [31] introduce RODNet, built upon 3D CNN layers with autoencoders, to extract motion information from the temporal dimension of RAM sequences. To extract information from radio frequency chirp sequences, Ref. [31] customize a module called M-Net, which consists of 3D CNN layers and max pooling layers. Arthur Ouaknine et al. [25] present a multi-view network for radar image segmentation, with a backbone network based on a dual encoder–decoder architecture. The multi-view architecture comes in two forms: MVA-Net, which uses 2D and 1D convolution layers to extract features from RAD, and TMVA-Net, which extends the temporal dimension by replacing the first block of 2D convolution layers in the encoder of MVA-Net with 3D convolution layers. Both networks achieve excellent semantic segmentation results in RA and RD views. Ravi Kothari et al. [26] explore fusion methods for the three radar information channels of RA, RD, and AD. They utilize RODNet-CDC [31] as the baseline model for RA information extraction and RAMP-CNN as the baseline for fusing the three radar channels of RAD. They further enhance the fusion effect through cross-attention mechanisms and center offset integration methods to achieve target detection.

LSTM [32] also exhibits good performance in extracting temporal information from radar images. Colin Decourt et al. [27] propose an encoder–decoder architecture called RECORD, which can leverage multi-view radar data for target detection. RECORD integrates convolutional operations with ConvLSTM to iteratively learn the implicit relationships of radar image sequences across different spatial and temporal dimension and the model utilizes past information stored in ConvLSTM’s memory to achieve causal detection. Fengde Jia et al. [33] propose a model for detecting and identifying targets in traffic scene data, using YOLOv8 as the basic framework and integrating ConvLSTM and attention mechanism modules. This model leverages ConvLSTM’s ability to process sequential data to extract temporal dimension information from RDM sequences, enhancing target recognition accuracy. Aman Shrestha et al. [34] introduce a human activity monitoring model based on recurrent LSTM and Bi-LSTM for action recognition by extracting human motion features from continuous time series of radar micro-Doppler or range-time information. The stacked Bi-LSTM network proposed in [34] is capable of capturing relevant motion constraints and features of human activities in continuous sequences along the temporal dimension, enhancing the model’s monitoring capabilities.

Temporal convolutional networks (TCN) [35], effective in capturing long-term contextual dependencies, has also found applications in tasks involving the extraction of temporal domain information. Compared to LSTM, TCN boasts a lighter structure and more efficient parallel computing capabilities, with its receptive field flexibly adjustable through settings for dilation coefficients and convolution kernel sizes. Karim Guirguis et al. [36] construct a network model using TCN and CNN to accomplish sound event localization and detection from sound spectrogram sequences. By pruning certain TCN modules, Ref. [36] manages to leverage the strengths of TCN while making the model even lighter, catering to the demands of low-power devices. Moritz Scherer et al. [37] combine 2D CNN with TCN to create a lightweight network for recognizing gesture types using RDM sequences. The 2D CNN module extracts spatial features from RDM, followed by the modified TCN to extract temporal information from the feature sequences. Compared to the TCN network in [35], Ref. [37] simplifies the proposed original network architecture, reducing the parameter size. As designed in [35], TCN networks are generally built using 1D CNN to extract features of a specific dimension. To directly process 3D data, Chunzhuo Wang et al. [38] propose a TCN architecture based on 3D CNN, which directly extracts eating action information from RD cubes and performs classification and recognition of eating actions. Three-dimensional TCN represents a novel attempt, demonstrating TCN’s ability to process data in multiple spatial and temporal dimension in parallel.

## 3. Materials and Methods

**Figure 1:**
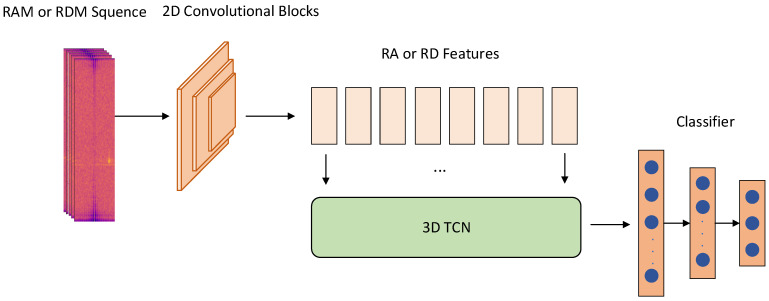
The architecture of RadarTCN.

RadarTCN comprises a spatiotemporal feature extraction module and a classification module, with its detailed structure illustrated in Figure 1. Temporal information from adjacent frames enhances the information richness of single-view radar images and the spatiotemporal feature distribution of these images is analyzed and presented in Section 3.1.1. Compared to 3D convolutional networks and 1D-TCN, the spatiotemporal feature extraction network proposed in this paper exhibits significant advantages in preserving information integrity. Additionally, it demonstrates superiority in parallel computing when compared to LSTM. These advantages are thoroughly analyzed and discussed in Section 3.1.2.

In Section 3.2, a down-sampling spatial feature extraction network with multi-layer max pooling is constructed. Section 3.2.1 introduces the application of the batch-normalization layer, aiming to handle irregular values in radar images and thereby ensuring the stability and training efficiency of the network. A multi-layer max-pooling down-sampling method is discussed in detail in Section 3.2.2. This is achieved by utilizing convolutional layers to focus on extracting image features without changing the data size and, simultaneously, employing multiple max-pooling layers to retain the maximum values and reduce the image size, thereby lowering data dimensionality and computational complexity without losing critical information.

In Section 3.3, an improved 3D-TCN network structure for extracting temporal information is presented. The methodology for designing a temporal feature extraction network using causal convolution, enabling online target classification, is elaborated in Section 3.3.1. The approach to achieve a lightweight design of 3D-TCN by pruning parts of the residual network structure is analyzed and demonstrated in Section 3.3.2.

In Section 3.4, a processing method based on simulated online radar data sequences from the CADDARA dataset is described in detail. To effectively prevent overfitting, a strategy involving the splitting of all scenario instances, shuffling of their order, and subsequent reintegration is adopted. Meanwhile, considering the simplification of the dataset compared to real-world traffic scenarios, data augmentation techniques are introduced to increase data complexity and enhance the model’s generalization ability.

In Section 3.5, the method and environment for model training are introduced. Meanwhile, a loss function suitable for training multi-label classification models is presented.

### 3.1. RadarTCN Architecture

The RadarTCN network architecture, designed in this paper, integrates spatial feature extraction modules, temporal feature extraction modules, and classification modules. It accurately extracts spatial distribution features and temporal variation features of targets of interest from radar image sequences and utilizes the classification module for final type recognition.

#### 3.1.1. Target Information in Radar Image Sequences

**Figure 2:**
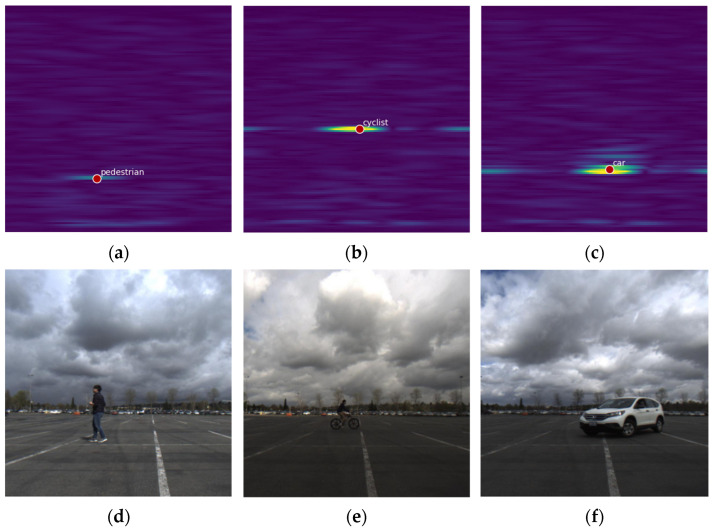
Distribution characteristics of target echoes on radar images from the CRUW dataset [12]. (**a**) Pedestrian on radar map; (**b**) cyclist on radar map; (**c**) car on radar map; (**d**) pedestrian on camera picture; (**e**) cyclist on camera picture; and (**f**) car on camera picture.

As discussed in Section 2.1, the spatial distribution of target echoes is greatly influenced by radar resolution and target size. With a determined radar resolution, the larger the target size, the more scattering points appear on the radar image. According to the characteristics of the FMCW radar system, the resolution of the radar in the range direction is independent of the target distance but the azimuth resolution is affected by the physical size of the antenna. The farther the target is, the worse the azimuth resolution and the wider the echo distribution of the target on the radar image. This is particularly evident in the Bird’s Eye View (BEV) image, which is converted from RAM. Figure 2 shows the echo distribution of pedestrians, cyclists, and vehicles on RAM and the corresponding comparison image of the camera. On the RDM, the distribution of a target in the range direction is related to its size, while its distribution in the Doppler direction is associated with short-term dynamic characteristics such as velocity. The size and motion state of the target determines its distribution shape on the RDM.

For a single RAM image, its echo distribution lacks dynamic information about the target, while a single RDM lacks target azimuth distribution information. To address this limitation, models like RAMP-CNN [24], TMV-Net [25], and Bivar Cross Atten [26] leverage multi-view RAD data to capture richer target echo features. However, the data size of RAD is significantly larger than that of RAM and RDM. In the CARRADA dataset, for instance, RAD has a dimension of (256, 256, 64), making it 64 times larger than RAM and 256 times larger than RDM. This high-dimensional input tensor increases both the parameter count of convolutional network models and computational complexity. Obtaining RAD data also demands more from the radar signal processing unit as it involves performing range-FFT, Doppler-FFT, and angle-FFT on the raw data. On the other hand, acquiring RA or RD data involves only two FFT operations. Consequently, using RAM or RDM data can greatly simplify the radar signal processing workflow, offering a more streamlined approach.

**Figure 3:**
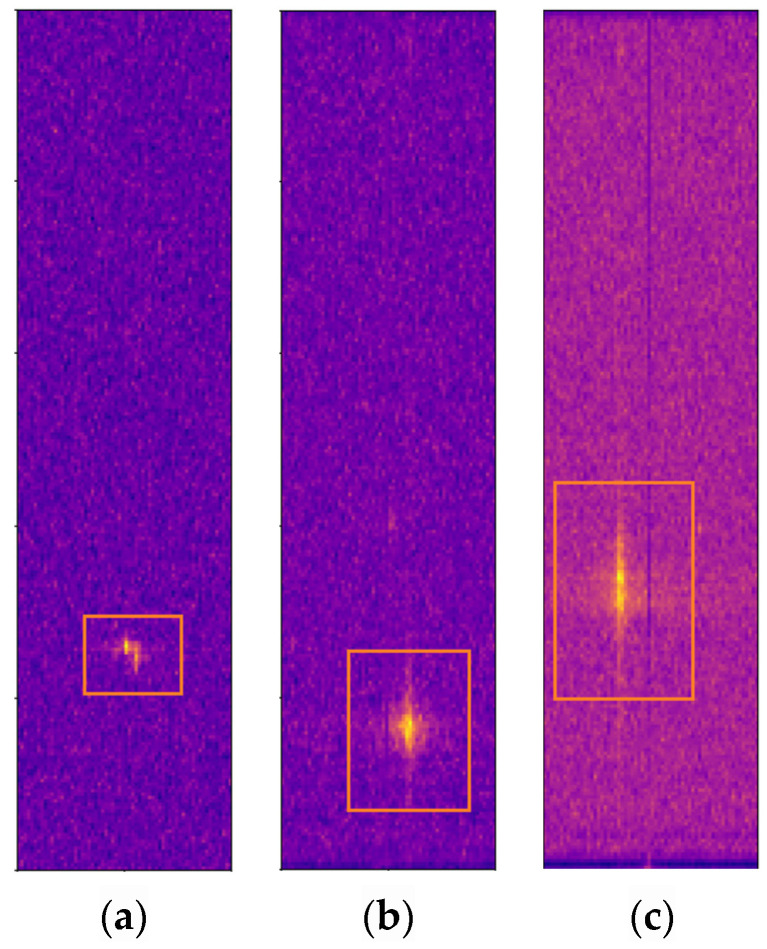
The variations in target echoes by stacking eight consecutive frames of RDM from the CARRADA dataset [11]. The echoes of all targets are circled by orange boxes. (**a**) Echo variations of a pedestrian in radar image sequences; (**b**) echo variations of a cyclist in radar image sequences; and (**c**) echo variations of a car in radar image sequences.

This paper utilizes radar image sequence data composed of consecutive RAM or RDM to overcome the information limitation inherent in a single radar image. Within the detection range of automotive radars, the data collected during the appearance of a target constitute a sequence spanning a certain duration, enabling the identification of different types of targets through the utilization of time-domain information. In radar image sequences, variations exist in the echo distribution of targets across adjacent periods. Changes in target distance between adjacent periods reflect alterations in target position. Moving cars exhibit greater positional changes than cyclists, who in turn show more positional variations than pedestrians. Additionally, variations in Doppler frequency between adjacent frames reveal the micro-motion characteristics of the target [39]. For instance, the swinging motion of pedestrians’ arms, the rotation of bicycle wheels, and the spinning of car wheels all generate micro-motion signatures that are superimposed on the Doppler frequency. These micro-motion features are attributes specific to certain types of objects and can assist models in identifying target types [40]. In RAM sequence data, changes in target positions between adjacent frames add temporal dynamic information. In RDM sequence data, variations in target radial distance and Doppler frequency can reflect the target’s horizontal state information. Figure 3 demonstrates the variation in target echoes across consecutive frames. By overlaying and displaying eight continuous frames of RDM (Range Doppler Map), it illustrates the spread of echoes from a pedestrian, a cyclist, and a car on the RDM.

#### 3.1.2. Spatial-Temporal Feature Extraction

To achieve precise recognition by extracting target feature information from both the spatial and temporal dimensions, a spatiotemporal feature information extraction module was formed by combining 2D convolutional blocks and 3D-TCN. Spatial distribution features are extracted from radar images using 2D convolutional blocks, resulting in 2D spatial feature maps. These maps, derived from adjacent frames, are then rearranged to construct a 3D cube that incorporates the temporal dimension. Subsequently, 3D-TCN is employed to extract information from the temporal dimension of this 3D cube.

TMV-Net [25] employs 3D convolutional layers in the convolutional section of the encoder branch to simultaneously handle temporal and spatial dimension information, aiming to extract spatiotemporal features. However, this approach may lead to the loss of temporal dimension information, as 3D convolutional layers lack the ability to capture long-term dependencies. The spatiotemporal feature information extraction module proposed in this paper separates the tasks of extracting spatial and temporal dimension information. Specifically, 2D convolutional blocks handle spatial dimension information, while 3D-TCN processes temporal dimension information. When 3D-TCN is employed to process the 3D data cube, it preserves the spatial features while exclusively operating along the temporal dimension. Additionally, 3D-TCN exhibits advantages in capturing multi-scale information through the introduction of a multi-scale strategy via temporal convolutional modules, enabling the extraction of both multi-scale and long-term dynamic information.

In [27], RECORD utilizes LSTM modules to achieve spatiotemporal feature extraction. However, due to the recurrent structure of LSTM, calculations can only be performed in temporal order, limiting its parallel computation efficiency and subsequently slowing down model training and inference speeds. The spatiotemporal feature information extraction module designed in this paper consists mainly of convolutional layers and its parallel computation pattern enhances the computational efficiency of model training and inference. Compared to the spatiotemporal feature extraction module in [37], after obtaining spatial features, TinyRadar uses the flatten function to merge the range and Doppler dimensions into a one-dimensional vector, which is then processed by 1D-TCN to extract temporal dimension information. This architecture is suitable for tasks involving gesture recognition as it focuses less on spatial information of the hand and more on temporal variations. However, this approach is unacceptable for traffic object recognition tasks because traffic objects exhibit a wide range of spatial features. In contrast, RadarTCN utilizes 2D convolution blocks to extract spatial information, which is then arranged into a spatial feature map sequence in temporal order. Following this, 3D-TCN extracts feature information in the temporal domain while preserving spatial features.

#### 3.1.3. Target Classification

Fully connected layers are chosen as the fundamental building blocks for constructing the classification network. The high-dimensional features from the spatiotemporal information processing network are fused and mapped to the fully connected layers. These layers are capable of processing input information from a global perspective, a characteristic that makes them particularly suitable for radar image data, effectively capturing key features. The classification module designed in this paper consists of three fully connected layers, which, through the judicious allocation of input and output feature channels, achieve reduced complexity and rapid classification.

### 3.2. Two-Dimensional Convolutional Blocks

**Figure 4:**
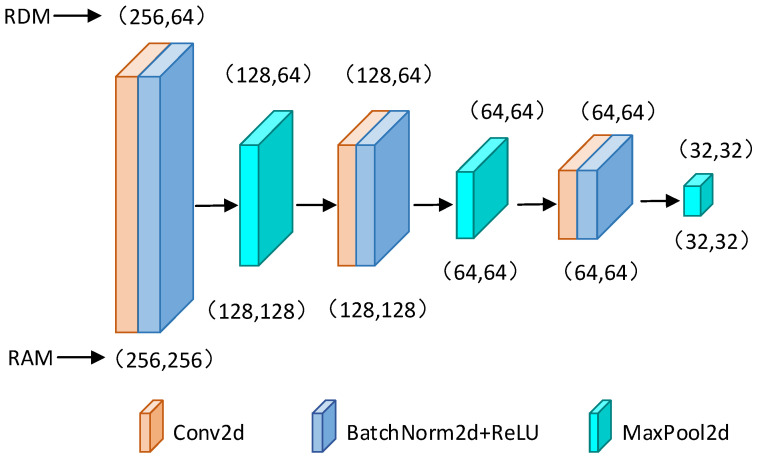
The architecture of the convolutional block group. RadarTCN_RA and RadarTCN_RD have different parameters in the first and second max-pool layers.

The radar image spatial feature extraction module comprises three 2D Convolutional Blocks, each containing a 2D convolutional layer followed by a batch-normalization layer and a ReLU activation function, which are then connected to a max-pooling layer. The structural diagram of the spatial feature extraction module is shown in Figure 4.

#### 3.2.1. RadarTCN_RA and RadarTCN_RD

Due to differences in the sizes of RDM and RAM, the convolutional blocks processing the RDM sequence and the RAM sequence have varying kernel sizes in some of the max-pooling layers, while the other structures remain the same. We name the RadarTCN that processes the RDM sequence as RadarTCN_RD and the one that processes the RAM sequence as RadarTCN_RA.

For RadarTCN_RD, the input tensor data size of the first 2D convolutional layer is (256, 64), where RDM has 256 sampling points in the range direction and 64 sampling points in the Doppler frequency direction. The kernel size of the 2D convolutional layer in each block is (3, 3) but the kernel sizes of the 2D max-pooling layers differ in each block. Specifically, the kernel sizes of both the first and second 2D max-pooling layers are (2, 1), while the kernel sizes of the third 2D max-pooling layer are (2, 2). When the input data are RAM data, the size of the RAM tensor is (256, 256) with 256 sampling points in the angular dimension, exceeding the size of RDM. In the RadarTCN_RA structure, to accommodate this larger RAM size, we adjust the 2D max-pooling layers within the 2D convolutional blocks: the kernel sizes of the first and second 2D max-pooling layers are changed to (2, 2), while the other network structures remain unchanged.

Radar echo signals inherently carry path interference and hardware circuit noise, potentially leading to negative values in specific positions of the spectrogram. This, in turn, can cause significant fluctuations in data values and may result in gradient vanishing or explosion during model training. To address this issue, a batch-normalization layer is incorporated following each 2D convolutional layer, normalizing the data. This approach accelerates the model’s convergence and enhances its stability during training. Additionally, using batch-normalization layers contributes to regularization, preventing model overfitting [41,42].

#### 3.2.2. Multi-Layer Max-Pooling down Sampling

Due to a large amount of raw radar image data input, a multi-layer max pooling down-sampling method is proposed to reduce the data size. The stride parameter of the 2D convolutional layers within the convolution blocks is set to 1 to ensure lossless extraction of spatial distribution feature information. By employing multiple 2D max-pooling layers, the method attains image size reduction and extracts maximum values, thereby effectively decreasing the computational complexity of the network while preserving valuable information. This achieves a dual optimization effect.

For the RadarTCN_RA spatial feature extraction network, the input RAM has 256 sampling points in the range direction and 256 sampling points in the azimuth direction, with each sampling point sized at 64 bits. The storage space occupied by the RAM is (256 × 256) × 64 bit = 4 Mbit. After passing through the max-pooling layer of the first 2D convolution blocks, the feature map size is reduced to (128 × 128) × 64 bit = 1 Mbit, representing a fourfold reduction in image size while preserving local features of the maximum echo values on the radar image. Similarly, the second and third 2D Convolution Blocks further reduce the radar image size by fourfold each, resulting in an output feature map size of 64Kbit for the spatial feature extraction network while retaining valuable spatial information.

Analogous to RadarTCN_RA, the RadarTCN_RD spatial feature extraction network reduces the input RDM from 1Mbit to 64Kbit while preserving valuable Doppler and range features.

**Figure 5:**
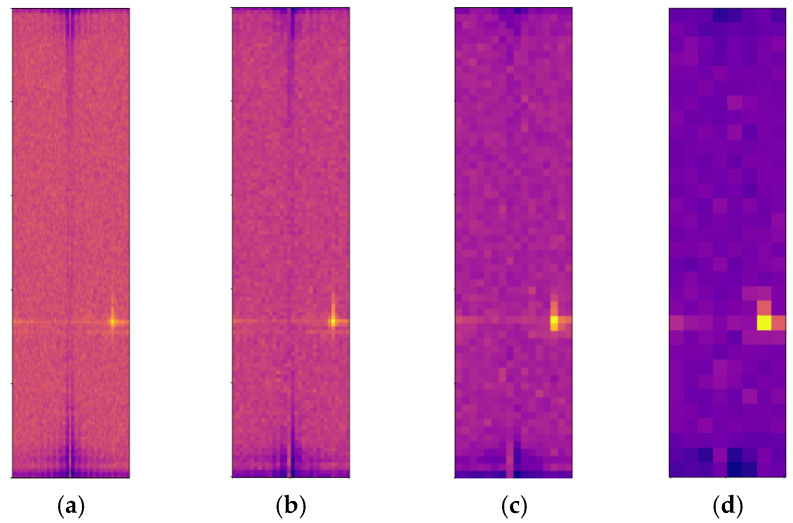
The effectiveness of max pooling layers in extracting RDM features from the CARRADA dataset [11]. (**a**) Original RDM; (**b**) RDM after undergoing one max-pooling layer; (**c**) RDM after undergoing two max-pooling layers; and (**d**) RDM after undergoing three max-pooling layers.

As illustrated in Figure 5, for RDM, the echo information related to the target signal is predominantly confined to a small region within the RDM graph. The brightest portion of this region denotes the most precise target location, corresponding to the highest values in the radar spectrogram. Conversely, areas outside this bright region primarily consist of background and interference signals, which are considered less valuable. To concentrate on the valuable regions within radar images and reduce the processed image size, Ref. [24] sets the stride parameter of the 3D convolutional layer of the CAE module to 2 to gradually reduce the image size and achieve a reduction in the amount of calculation and parameter scale. Nonetheless, this approach of increasing the convolutional stride may result in the loss of the maximum value, thereby posing a risk of discarding valuable information.

### 3.3. 3D-TCN

**Figure 6:**
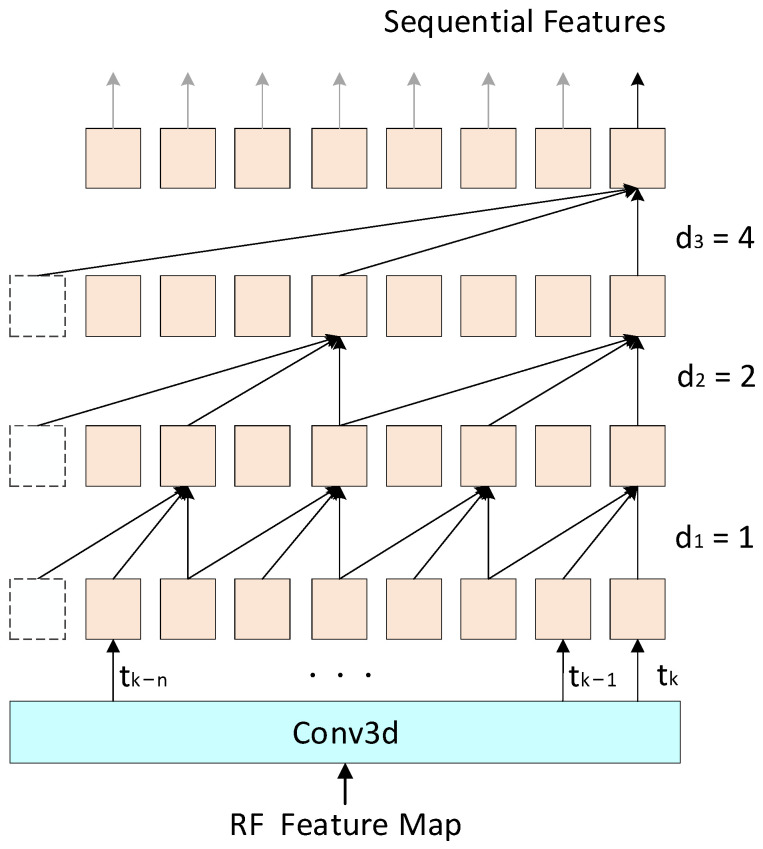
The framework of 3D-TCN.

As shown in Figure 6, the 3D-TCN proposed in this paper consists of a 3D convolutional layer and three residual blocks. Within each residual block, there is a 3D dilated causal convolution followed by a ReLU activation function. Additionally, a shortcut connection, comprising a 1 × 1 Conv3d, aids in feature mapping and dimension adaptation. The output of the residual block is obtained by summing the outputs from the convolutional path and the shortcut connection. The parameters of the layer are presented in Table 1.

#### 3.3.1. Online Targets Classification

It is worth noting that the causal convolution, designed specifically for processing sequential data, produces outputs based solely on current and past inputs, without being influenced by future data, as shown in (3) as follows:

(3) $$p\left(t\right)=\overset{T}{\underset{t=1}{\prod }}p\left(x_{t}|x_{1},\cdots ,x_{t-1}\right)$$

where p(t) represents the output result of the causal convolution at time t, $x_{t}$ represents the input at time t, and $\;x_{1},\cdots ,x_{t-1}$ represent the input data from previous time steps.

The 3D-TCN architecture proposed in this paper differs from the Eat-Radar 3D-TCN architecture described in [38]. Eat-Radar 3D-TCN consists of nine residual blocks and has two structural types: shape variant and shape invariant. Additionally, Eat-Radar 3D-TCN does not employ causal convolution. For input data with a sequence length of n, the output at time $t_{k}$ is influenced by input data from time $t_{k+1}$ to $t_{k+n}$. This suggests that the Eat-Radar 3D-TCN architecture can only perform action recognition using offline data and is incapable of online recognition. In contrast, the 3D-TCN proposed in this paper uses dilated causal convolution, allowing flexible expansion of the model’s temporal receptive field while maintaining online recognition capabilities.

The three residual blocks designed in this paper have the same structure, including causal convolution and dilated convolution. The kernel sizes of the dilated causal convolution are (3, 1, 1). The 3D cube is represented in the form of (frames, range, and Doppler) and we only apply dilated and causal convolution calculations along the frames dimension, while the range and Doppler dimensions remain unchanged. Therefore, the dilation coefficient of the dilated causal convolution is $\left(d_{l},\;1,\;1\right)$, where $d_{l}=2^{l-1}\;\left(1\leq l\leq 3\right)$ and l represents the layer number. The input data sequence length for the first residual module is N and the output of the 3D-TCN at time $t_{k}$ is solely dependent on the existing input frames from time $t_{k-1}$ to $t_{k-\left(N-1\right)}$. This allows RadarTCN to possess the capability of online classification.

#### 3.3.2. Lightweight Residual Block

**Figure 7:**
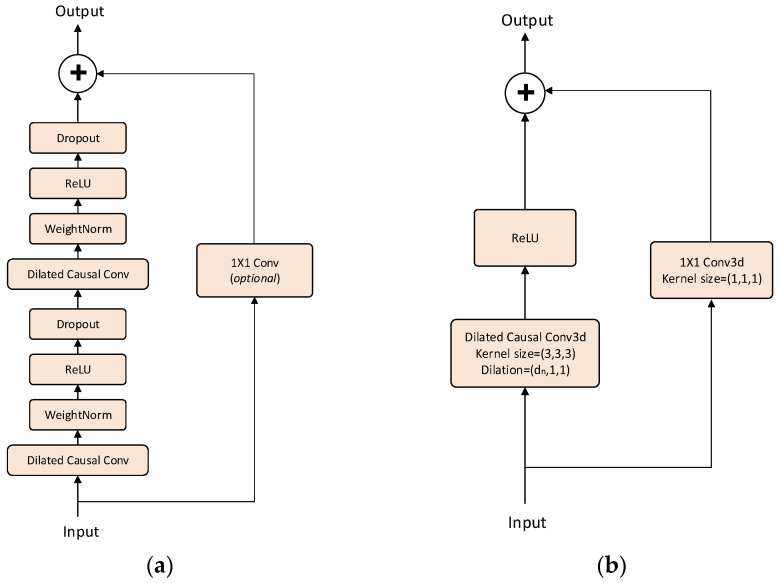
Simplification of residual network. (**a**) The proposed TCN residual block is in [35]. (**b**) The simplified residual block of 3D-TCN.

In the radar image spatial feature extraction phase, a sequence of spatial feature maps is outputted. These maps are then stacked temporally to form a 3D cube. To adjust its dimensions, a 3D convolutional layer is employed in the input section of the 3D-TCN. Within the residual blocks, convolutional operations are performed using 3D convolutional layers to capture spatial information from the sequences. The TCN residual network proposed in the literature [35] comprises two layers of dilated causal convolution, as depicted in Figure 7 Three-dimensional convolutional layers that process three-dimensional data require a large number of parameters and demand high computational resources. In multi-layer TCNs, using too many 3D dilated causal convolution layers can result in a complicated network structure, thereby heightening the risk of overfitting. 

Compared to the TCN residual blocks described in [35], those in this paper undergo structural simplification. Specifically, dropout layers, weight-norm layers, and the second dilated causal convolution layer are pruned, leaving only a single layer of dilated causal convolution and the ReLU activation function. These simplifications significantly reduce model complexity and parameter size, resulting in a lightweight network.

### 3.4. Radar Image Sequence Dataset

The CARRADA dataset comprises outdoor scenes jointly collected using am FMCW Radar and a camera. It encompasses RAD data, RAM data, RDM data, camera images, and their corresponding annotations. For the purpose of model training and validation, this study specifically employs RAM and RDM data. Hence, the CARRADA dataset has been selected for the rigorous verification and evaluation of RadarTCN.

The CARRADA dataset comprises 30 sequences, 78 outdoor scene instances, and 7193 annotated frames of data. The scene instances include a single pedestrian walking in a preset pattern, a cyclist moving along a designated path, vehicles approaching and moving away, the simultaneous appearance of pedestrians and vehicles, cyclists and cars moving together along the road, and two vehicles or two pedestrians moving simultaneously on the road, among others. Each individual scene instance consists of multiple consecutive frames.

Given that RadarTCN relies on continuous frame sequences as input data, the CARRADA dataset must undergo splitting and packaging into numerous sequences. The sequence length is N, where N takes values of (3, 5, 8, 12, and 16). Data are selected from time point $t_{k}$ and the previous N−1 consecutive time points and a radar image sequence is constructed in chronological order. These sequences overlap by N−1 frames to ensure data continuity. Following this approach, RAM and RDM sequence datasets are constructed.

During the training process, if consecutive sequences all originate from the same scene instance, the risk of overfitting increases. To avoid this issue, it is necessary to split the scene instance data. All scene radar image sequences should be numbered and a list containing all sequence numbers should be generated. The sequence numbers in this list are randomly shuffled and they are subsequently divided into a training set and a validation set. This strategy aims to ensure diversity and effectiveness in model training. Considering that the scenarios encompassed by the CADDARA dataset seem simplified in comparison to real-world traffic situations and due to the potential risk of underfitting arising from the limited data available, data augmentation techniques are employed in the training dataset as a means of mitigation. Within the same sequence, image arrays are flipped, which not only ensures the continuity of adjacent frames but also effectively increases data complexity, thereby enhancing the model’s generalization ability.

The test set is constructed before the division of training and validation sets and it consists of partial data extracted from multiple scene instances. During the testing phase, to assess the model’s online target classification capability, radar data are simulated by integrating current radar images with nearby frames from previous moments, forming an input sequence. If the sequence covers an extended period, data from prior moments are temporarily held in a designated cache. With the advent of new testing cycles, the nearby frames in temporary storage undergo updates based on the aforementioned sequence assembly strategy, thereby guaranteeing data freshness and pertinence.

### 3.5. RadarTCN Training

Using the radar data sequences obtained from Section 3.4, with sequence lengths set to 3, 5, 8, 12, and 16, respectively, the RadarTCN_RD model and the RadarTCN_RA model are trained separately. The training batch size is set to 16 and the number of epochs is set to 100. During the training of RadarTCN, Adam is used as the optimizer and the weight decay parameter is set to $1.0e^{-2}$. The initial learning rate for the model is set to $1.0e^{-3}$ and it decays exponentially with a factor of 0.9 every five training epochs to find the optimal model parameters. The model architecture is built using PyTorch and the training and validation processes are conducted on an NVIDIA Quadro RTX 2060 GPU.

Due to the presence of multiple targets in single-frame images within the instance sequences of the CARRADA dataset, multi-label classification tasks need to be performed. During the model training phase, multi-label soft margin loss is chosen as the loss function to accommodate multi-label support. Additionally, the dataset exhibits an imbalanced distribution of target sample categories, which can lead the model to favor the majority class. To mitigate this issue, weight factors were incorporated into the loss function for each category of data.

(4) $$loss\left(x,y\right)=-\frac{1}{C}\times \underset{i}{\sum }w_{i}y_{i}\times \log\left({\left(1+\exp\left(-x_{i}\right)\right)}^{-1}\right)+\left(1-w_{i}y_{i}\right)\times \log\left(\frac{\exp\left(-x_{i}\right)}{1+\exp\left(-x_{i}\right)}\right)$$

where $w_{i}$ denotes the weight of the i-th category, which also represents its proportion in the dataset. $y_{i}$ represents the ground truth, $x_{i}$ represents the predicted value, and C represents the total number of categories.

## 4. Results and Discussion

In Section 4.1, the model proposed in this paper is tested and verified using the simulated radar online data obtained from Section 3.4. Precision, recall, accuracy, and AP are used to evaluate the target classification ability of RadarTCN, while parameters and GMACS are used to assess both the lightweight nature and the computational complexity of the RadarTCN network. When the length of the radar image sequence is 8, the evaluation results are presented in Table 2. RadarTCN exhibits the capability to perform online target classification on both RAM and RDM sequences, achieving a comprehensive classification accuracy of approximately 98% while maintaining a relatively small parameter size and low computational complexity.

As shown in Table 3, comparing the evaluation results of RadarTCN with other classification models on the CARRADA dataset reveals several key findings. RadarTCN achieves high classification accuracy while having a significantly smaller parameter size and lower computational complexity compared to RAMP-CNN [24] and the Bivar Cross Atten [26], which use 3D convolution to process multi-view RAD data. This demonstrates the advantages of RadarTCN’s network architecture, which separates the processing of spatial and temporal information in preserving the integrity of spatiotemporal data. Furthermore, it indicates that RadarTCN’s use of multi-layer max pooling for down sampling, as well as its lightweight design approach for 3D-TCN, significantly reduces both parameter size and computational complexity.

**Figure 8:**
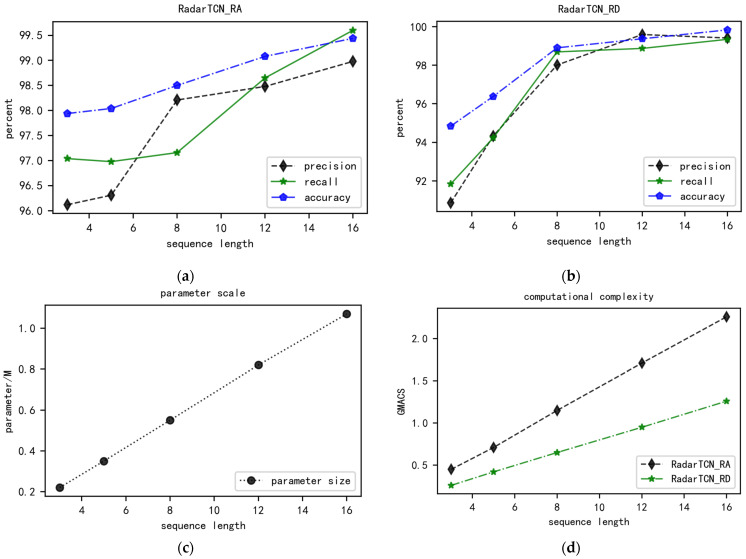
The experimental results of different sequence lengths. (**a**) The curve of RadarTCN_RA’s classification performance varying with sequence length; (**b**) the curve of RadarTCN_RD’s classification performance varying with sequence length; (**c**) the variation in RadarTCN’s parameter size with sequence length; and (**d**) the variation in RA’s GMACS with sequence length.

In Section 4.2, Figure 8 demonstrates how sequence length affects the performance of RadarTCN. Experimental results indicate that an increase in temporal information can enhance the model’s classification capability but a larger input data size also leads to a linear increase in computational complexity and parameter scale.

In Section 4.3, the ablation experiment results indicate that:

- Disrupting the integrity of the spatial feature extraction module leads to a decrease in classification metrics;
- Using 1D-TCN instead of 3D-TCN results in a loss of spatiotemporal information;
- The information richness of a single radar image is lower compared to radar image sequences.

### 4.1. Evaluation and Comparison

An evaluation of RadarTCN_RA and RadarTCN_RD is conducted using instance sequences from the same scenarios. These radar data sequences consist of targets such as backgrounds, pedestrians, cyclists, and automobiles. The actions of the targets in these scenarios include movements like moving away and approaching, as well as lateral shifts from left to right or vice versa.

The performance of the models is evaluated with a radar image sequence length set to 8 and the results are presented in Table 2. RadarTCN_RA achieves a classification accuracy of 98.65%, a recall rate of 97.89%, and a precision rate of 98.52%. Meanwhile, RadarTCN_RD attains a classification accuracy of 98.69%, a recall rate of 98.01%, and a precision rate of 98.90%. RadarTCN demonstrates high classification performance metrics on both RAM and RDM data.

In terms of parameter size, RadarTCN has only 0.55 million parameters. Additionally, regarding the computational complexity metric [43], RadarTCN_RA has a GMACS of 1.15, while RadarTCN_RD has a GMACS of 0.65. With its small parameter size and low computational complexity, RadarTCN qualifies as a lightweight model.

Both RODNet-CDC and RadarTCN_RA use RAM as input data. Compared to RODNet-CDC, RadarTCN_RA achieves an 11 percentage point improvement in the AP metric. It also reduces the number of parameters by nearly 90% and computational complexity by a factor of more than 19. The AP of RAMP-CNN and the Bivariate Cross Attention Network is 89% and 94%, respectively, while RadarTCN_RA and RadarTCN_RD can reach 99%. This represents a relative improvement of 5 percentage points in the AP metric compared to the Bivariate Cross Attention Network. In terms of parameter size and computational complexity, RadarTCN offers even more advantages: a reduction of over 20 times in the number of parameters and a reduction of over 28 times in GMACS.

This comparative test demonstrates the advantages of the RadarTCN architecture. RODNet-CDC, RAMP-CNN, and Bivariate Cross Attention Network primarily utilize 3D convolutional layers to extract spatial and temporal feature information from targets, whereas RadarTCN_RA employs a feature extraction network that separates the spatial and temporal domain, effectively avoiding information loss. Additionally, RadarTCN_RA utilizes 2D convolutional blocks to efficiently reduce the size of radar images, decreasing both parameter size and computational complexity. Furthermore, while RAMP-CNN and Bivariate Cross Attention Network require RAD as input data, RadarTCN_RA only needs RAM data, and RadarTCN_RD only requires RDM data, reducing the system’s demands for the radar signal processing frontend.

Compared to the experimental results in [26], the RadarTCN model proposed in this paper achieves optimal classification performance on the CARRADA dataset while also excelling in model lightweighting.

### 4.2. Results of Different Sequence Lengths

By modifying the length of the input data sequence for the model and observing the test results, the findings are presented in Figure 8. As the input data sequence length increases from 3 to 16, the classification metrics of precision, recall, and accuracy for both RadarTCN_RA and RadarTCN_RD gradually rise. Moreover, the experimental results indicate that the longer the radar sequence, the better the recognition performance of RadarTCN.

The test results presented in Figure 8a,b indicate that, when the length of the input data sequence is no more than 8, the evaluation metrics of RadarTCN_RD are significantly affected by the sequence length. This is due to the incorporation of micro-motion feature information in the RDM sequence compared to the RAM sequence, making the classification performance of RadarTCN_RD more sensitive to the length of the radar image sequence. In contrast, the evaluation metrics of RadarTCN_RA exhibit a slow-increasing trend with the sequence length. However, when the input data sequence length exceeds 8, the changes in the classification evaluation metrics for both RadarTCN_RD and RadarTCN_RA become relatively flat.

Figure 8c demonstrates the variation in model parameter sizes for RadarTCN_RA and RadarTCN_RD with respect to the length of the input data sequence. Notably, the model parameter size is positively correlated with the input sequence length. When the length of the radar image sequence increases to 16, the parameter size of the RadarTCN model reaches 1.07 million, which is still significantly lower than those of other models proposed by researchers, as listed in Table 3.

Meanwhile, Figure 8d exhibits the curves of computational complexity for RadarTCN_RA and RadarTCN_RD as a function of sequence length. Specifically, GMACS increases proportionally with sequence length. When the sequence length increases to 16, the highest GMACS value reached by RadarTCN_RA is 2.26, whereas for RadarTCN_RD it is 1.26.

Considering both classification performance and model lightness, the optimal input sequence length for achieving the best overall model performance is determined to be 8.

### 4.3. Ablation Studies

In Section 4.1, the overall architecture of RadarTCN is presented. In this section, modifications are made to parts of the RadarTCN structure and the impact of these modifications on the overall algorithm’s recognition capability is observed.

- Remove the batch-normalization layer from the convolutional blocks

Train and test RadarTCN_RA and RadarTCN_RD without the batch-normalization layer on the CARRADA dataset. The obtained classification precision, recall, and accuracy are shown in Table 4. The experimental results demonstrate that removing the batch-normalization layer significantly degrades the classification performance of RadarTCN. This indicates that the batch-normalization layer is an essential component of RadarTCN.

- Substitute 3D-TCN with 1D-TCN in the model architecture

Once spatial features are extracted, stack them along the temporal dimension to form a three-dimensional feature matrix. Subsequently, employ the flatten function to merge the temporal and spatial dimensions, thereby allowing the utilization of 1D-TCN for target feature extraction. Upon training and evaluation on the CARRADA dataset, the test results, presented in Table 5, reveal a notable decline in performance. Specifically, when compared to the RadarTCN_RD model utilizing 3D-TCN, precision drops by 11.11%, recall decreases by 13.17%, and accuracy diminishes by 6.75%. This substantial degradation in recognition performance indicates that employing 1D-TCN for extracting spatiotemporal features from sequential data entails a significant loss of information.

- Replace the input radar image sequence with a single radar image

The models RadarTCN_RA and RadarTCN_RD only take a single radar image as the input. They disregard the temporal information between consecutive frames and rely solely on the spatial distribution of the single radar image for classification. The evaluation results are presented in Table 6. Compared to the scenario where the models input a radar image sequence of length 8, the recognition performance of both RadarTCN_RA and RadarTCN_RD significantly decreases when only a single radar image is used. A more substantial drop in performance is observed for the RadarTCN_RA model. A comparative analysis with Table 2 reveals that temporal information in radar sequence data can significantly compensate for information deficiencies in RA or RD data. Thus, it effectively improves the accuracy of the models in target classification based on RA or RD data.

## 5. Conclusions

In this paper, the semantic information of targets in radar images, encompassing spatial distribution and temporal dynamics, is explored. Leveraging these features, RadarTCN, a target recognition framework that integrates 2D CNN and 3D-TCN for effective recognition, is introduced. This model uniquely achieves online recognition using radar sequence data from RA or RD. Its small parameter size, low computational complexity, and hardware adaptability facilitate practical implementation and widespread use. On the CARRADA dataset, RadarTCN demonstrates superior performance in classifying cars, bicycles, and pedestrians compared to other network models.

Compared to actual traffic scenarios, the scenarios covered by the CARRADA dataset are relatively simple. To improve the performance of the model proposed in this paper in actual traffic scenarios, further training and validation using real traffic datasets are needed. The more working conditions covered by the training dataset, the more robust the trained model will be.

## Figures and Tables

**Table 1:** The architecture of 3D-TCN and the output data size of each layer.

Layer	Output ^1^	Dilation
Input	16 × 8 × 32 × 32	-
Conv3d	16 × 8 × 16 × 16	-
Residual block1	16 × 8 × 16 × 16	(1, 1, 1)
Residual block2	16 × 8 × 16 × 16	(2, 1, 1)
Residual block3	16 × 8 × 16 × 16	(4, 1, 1)

^1^ The sequence length is 8.

**Table 2:** The experimental results of RadarTCN_RA and RadarTCN_RD.

Model	Precision (%)	Recall (%)	Accuracy (%)	Params (M ^2^)	GMACS
RadarTCN_RA ^1^	98.65	97.89	98.52	0.55	1.15
RadarTCN_RD ^1^	98.69	98.01	98.90	0.55	0.65

^1^ The sequence length is 8. ^2^ M stands for million.

**Table 3:** Performance comparison between different models.

Model	Input	AP (%)	Params (M ^2^)	GMACS
RODNet-CDC [31]	RA	88	4.6	22.02
RAMP-CNN [24]	RAD	89	14.23	46.73
Bivar Cross Atten [26]	RAD	94	11.05	32.45
RadarTCN_RA ^1^	RA	99	0.55	1.15
RadarTCN_RD ^1^	RD	99	0.55	0.65

^1^ The sequence length is 8. ^2^ M stands for million.

**Table 4:** The experimental results without batch-normalization of RadarTCN_RA A and RadarTCN_RD.

Model	Precision (%)	Recall (%)	Accuracy (%)
RadarTCN_RA ^1^ without batch-normalization	33.33	16.36	61.53
RadarTCN_RD ^1^ without batch-normalization	17.37	33.33	63.85

^1^ The sequence length is 8.

**Table 5:** The results of the RadarTCN model using 1D-TCN.

Model	Precision (%)	Recall (%)	Accuracy (%)
RadarTCN_RA ^1^ using 1D-TCN	96.02	95.14	97.34
RadarTCN_RD ^1^ using 1D-TCN	87.57	84.34	92.15

^1^ The sequence length is 8.

**Table 6:** The results of RadarTCN using a single radar image as input.

Model	Precision (%)	Recall (%)	Accuracy (%)
RadarTCN_RA ^1^ with single RAM input	75.17	60.36	79.10
RadarTCN_RD ^1^ with single RDM input	85.69	87.82	90.84

^1^ The sequence length is 8.

## Data Availability

The data and the code of this study are available from the first author upon request.
